# Evaluation of Five Medicinal Plant Extracts on *Aphis craccivora* (Hemiptera: Aphididae) and Its Predator, *Chrysoperla carnea* (Neuroptera: Chrysopidae) under Laboratory Conditions

**DOI:** 10.3390/insects11060398

**Published:** 2020-06-26

**Authors:** Samy M. Sayed, Saqer S. Alotaibi, Nevien Gaber, Sayed-Ashraf Elarrnaouty

**Affiliations:** 1 Department of Economic Entomology and Pesticides, Faculty of Agriculture, Cairo University, Giza 12613, Egypt; ashrafelarnaouty@yahoo.com; 2 Department of Biotechnology, Faculty of Science, Taif University, Taif 888, Saudi Arabia; saqer-20@hotmail.com; 3 Plant Protection Research Institute, Agricultural Research Center, Dokki, Giza 12619, Egypt; gaberbio@yahoo.com; 4 Department of Biology, Faculty of Science, Princess Nourah bint Abdulrahman University, Riyadh 11671, Saudi Arabia

**Keywords:** biopesticide, biological control, botanical insecticides, lacewing, integrated pest management

## Abstract

Botanical insecticides that degrade rapidly are safer than persistent synthetic chemical insecticides, less harmful to the environment, decrease production costs and are not likely to cause insecticide resistance among pests. This study aimed to evaluate the effect of five different botanical extracts on the bean aphid, *Aphis craccivora* and the 2nd larval instar of the green lacewing, *Chrysoperla carnea* under laboratory conditions. Also, the flavonoids in the methanolic extracts of these tested plants were detected using HPLC analysis. The data from the HPLC analysis indicated that the tested plants differed in their flavonoid components. The total flavonoids were 869.4, 1125.6, 721.4, 1667.8 and 2025.9 mg/kg in *Psiadia penninervia*, *Salvia officinalis*, *Ochradenus baccatus*, *Pulicaria crispa* and *Euryops arabicus*, respectively. Moreover, there were many variations among these plants in the amount of each compound. The lethal concentration (LC_50_) value of *P. penninervia* extract on aphids was the lowest among all of the plants (128.546 µg/mL) followed by *O. baccatus* (626.461 µg/mL). Also, the LC_50_ value of *P. penninervia* extract on the 2nd larval instar of *C. carnea* (232.095 µg/mL) was significantly lower than those of all other four plant species extracts, while the other four plants did not show significant differences among them according to relative median potency analyses. Accordingly, *O. baccatus* extract had a strong effect on aphids and was safest for the predator. This finding suggests that *O. baccatus* could be exploited and further developed as an effective plant extract-based insecticide to be utilized in integrated pest management (IPM) programs against *A. craccivora*.

## 1. Introduction

Plants produce secondary metabolites and chemical substances to protect themselves from the attack of pests and pathogens [1]. There is a growing need for new active substances and products for pest control that decrease the unfavorable impacts of chemical insecticides on the environment and especially on human health [2]. Thus, thousands of plant extracts have been evaluated as alternatives to chemical insecticides. The use of botanical insecticides can cause mortality, infertile adults, slow growth, and a decrease in the egg viability of insects. However, these botanical extracts are generally less harmful to the environment, low cost, and their use as food ingredients indicate their low toxicity to humans [3,4]. Botanical aphicidal agents biodegrade naturally and are less persistent than synthetic chemical insecticides. Many studies have attempted to improve plant-derived aphicidal agents, and various biologically active compounds that are toxic to insect pests have been isolated and identified [5]. The Asteraceae (Compositae) family is considered as one of the most widespread families of flowering plants and it includes 1620 genera [6]. Numerous species of *Pulicaria* genus are rich in several botanical compounds such as flavonoids, isocomene, acetylenes, monoterpenes and sesquiterpene lactones. Moreover, several *Pulicaria* spp. have conventionally been used to repulse insects [7]. Also, the *Euryops* genus belongs to the Asteraceae family and includes 97 species. *Euryops arabicus* is widely distributed in Saudi Arabia and Yemen [8]. The genus *Psiadia* belongs in the aster tribe of Asteraceae family and consists of about 60 species growing in subtropical and tropical regions of Asia and Africa. Many investigations on the pharmacology and phytochemistry of this genus have been conducted and have resulted in the isolation and identification of 73 compounds including flavonoids, terpenoids, coumarins and phenylpropanoids [9]. The genus *Salvia* (sage) is the most widespread of the genera of the Lamiaceae family, containing more than 900 species throughout the world [10]. *Salvia officinalis* L. is the most well-known species of this genus and is commonly known as red or garden sage. It is native to Mediterranean regions and is one of the most popular medicinal plants in Arabian countries [11]. *Ochradenus baccatus* (Family: Resedaceae) is a medicinal shrub that is widely distributed in desert regions of the North Africa and Middle East. Moreover, it is very rich in glucosinolates, the level of which depends on the plant part [12].

The use of chemical pesticides can harm biocontrol agents and result in pest resurgence and/or outbreaks. Therefore, integrated pest management (IPM), which combines biopesticides and biocontrol agents is receiving more attention as an ecologically safe management strategy under which predators and parasitoids are combined with botanical extracts [13].

However, for pest control, it is important to determine the effects of the plant substances on the natural enemies. Different investigations have been carried out to estimate the effect of botanical extracts on insect pests and biocontrol agents. For example, the combination of the entomopathogenic fungi and botanical extracts (eucalyptus or neem) caused a large reduction in the fecundity and survival of the wheat aphid, *Sitobion avenae* [14]. Some leaf extracts of *Chromolaena odorata* (Family: Asteraceae) have been ranked as harmless while others were moderately harmful to the egg parasitoid, *Trichogramma japonicum* [13].

The bean aphid, *Aphis craccivora* Koch (Hemiptera: Aphididae) infests the leaves, twigs, pods and flowers of the bean plant. Also, this aphid species causes losses of up to 100% in the yield of various legumes species [15]. Moreover, *A. craccivora* transmits approximately 20 nonpersistent plant viruses in different regions of the world. The use of synthetic chemical pesticides to control this aphid on a large-scale has resulted in environmental hazards and toxic effects on the beneficial organisms, especially natural enemies [16]. These side effects have stimulated research on alternative strategies of aphid management. The alternative methods to synthetic pesticides for aphid control include using biodegradable and natural compounds, such as plant extracts, flavonoids, saponins, lectins and essential oils [17]. The number of studies investigating the potential role of plant-derived essential oils (EOs) in aphid control has showed a notable increase over the last 20 years. EOs can be extremely effective in pest control and thus, should be seriously considered as environmentally friendly aphicides [18].

The green lacewing, *Chrysoperla carnea* (Stephens) (Neuroptera: Chrysopidae) is considered as a generalist predator. Its larvae actively predate on different species of soft bodied insect pests such as aphids, thrips, whiteflies, mealy bugs and coccids [19].

This study was carried out to evaluate five different botanical extracts against the bean aphid, *A. craccivora* and its biological control agent, the 2nd larval instar of the green lacewing, *C. carnea* under laboratory conditions. Due to the importance of flavonoids in plant protection from insect and microbial attack [20], HPLC analysis was conducted to determine the flavonoid components of five medicinal plants.

## 2. Materials and Methods

### 2.1. Plant Samples and Extracts

Fresh leaves of five medicinal plant species were collected in May 2019 from their natural habitat in the Al-Shafa region, Taif Provenance, as indicated in Table 1. The collected fresh leaves (1 kg from each plant species) were ground to fine powder in liquid nitrogen by a mortar and pestle. Extraction and dissolution were carried out [21] with some modifications. Five grams (fine powder) of each sample was extracted with 100 mL 95% methanol at 35 °C for two days in a thermostat water bath shaker. After cooling, each extract was centrifuged at 7000 rpm for 15 min and filtered with Whatman filter paper No.1 and this was repeated three times to get a pure supernatant. Then, the supernatant was passed through a Buchner funnel in a rotary vacuum evaporator under a temperature of 30 °C. During evaporation, the total extract of each plant was adjusted to one liter. Then, 5 mL of each extract was taken and stored for HPLC analysis and the evaporation was completed. Each extract (pellet) was dissolved in aqueous solution of dimethylsulfoxide 1% (DMSO) with a final concentration of 50 mg/mL). The plant extracts were stored at 4 °C until they were used for the bioassays.

### 2.2. HPLC Analysis and Quantification of Phenolic Compounds

The analysis and detection of phenolic compounds for the five tested plants were carried out according to [22] with some modifications using Agilent 1260 infinity HPLC Series (Agilent, Santa Clara, CA, USA), equipped with a quaternary pump. The column used was a Kinetex^®^ 5 µm EVO C18 100 mm × 4.6 mm, (Phenomenex, Torrance, CA, USA) and operated at 30 °C. The separation was conducted using a ternary linear elution gradient with (A) HPLC grade water 0.2% and H_3_PO_4_ (*v*/*v*), (B) methanol and (C) acetonitrile. Then, a volume of 20 µL was injected. AVWD detector set at 284 nm was used for detection of phenols and flavonoids. The analysis was done at the Food Safety and Quality Control Laboratory, Faculty of Agriculture, Cairo University (FSQC 0911-0915/2019).

### 2.3. Insects

The tested insects were obtained by rearing at the laboratory for mass production of predators at the Faculty of Agriculture, Cairo University. The 2nd larval instar of the green lacewing, *C. carnea* and one-day adults of the cowpea aphid, *A. craccivora* were used in this study.

#### Bioassay

Each extract was diluted to obtain four concentrations of 1000, 500, 250 and 125 µg/mL. The solvent (1% aqueous DMSO) was used as the control. An amount of 3 mL from each extract/concentration was sprayed with equal homogeneity by a fine sprayer machine (Thomas Scientific, USA) on 300 cm^2^ (10 µL/cm^2^). This area contained 32 leaves of broad bean plant that were divided into 2 groups (16 for each group). Each leaf from the first group had 5 aphid individuals while each leaf from the second group had 5 predator individuals (2nd larval instar) with about 30 individual aphids as prey. The spray was applied directly onto the plant leaves containing the tested insects. After the spray application, each leaf was placed in a Petri dish (100 × 15 mm) with moistened cotton tissues to maintain humidity. Then, the dishes were kept in a plant growth chamber at 25 ± 1 °C, 65 ± 3 RH and 14:10 L:D. Four leaves from each group were considered as one replicate. Accordingly, each replicate contained 20 individual aphids or predators, with a total of 4 replicates per concentration. After 24 h, aphid and predator mortality were assessed by gentle probing with a fine brush and observing the lack of predator movement and the change in the aphid’s body to a post-mortem color [23].

### 2.4. Statistical Analysis

The mortality rates in the treatments were corrected with that in the control according to Abbott’s formula [24]. The lethal concentrations (LC_50_) were estimated using Log-probit analysis of mortality versus concentration. Then, significant differences between the LC_50_ values were determined by estimating the confidence intervals of the relative median potency. Differences among LC_50_ values were judged as statistically significant when 1.0 was not found in the 95% confidence interval of relative median potency. All statistical analysis were conducted by SPSS software program, version 23 [25].

## 3. Results

The data obtained from the HPLC analysis for the five medicinal plants indicated that these plants differed in their component of flavonoids. The total flavonoids were 869.4, 1125.6, 721.4, 1667.8 and 2025.9 mg/kg in *P. penninervia*, *S. officinalis*, *O. baccatus*, *P. crispa* and *E. arabicus*, respectively (Table 2). Also, some compounds were detected in high amounts in one or more of the tested plants but the same compounds were not detected in other tested plants, for examples, gallic acid and benzoic acid. Moreover, although the three plants *P. penninervia*, *P. crispa* and *E. arabicus* belong to the Asteraceae family, they contained different flavonoids and different concentrations of total flavonoids.

Table 3 presents the LC_50_ values of the five tested extracts on the aphid, *A. craccivora*. The LC_50_ value for the *P. penninervia* extract was the lowest of all tested plants with a LC_50_ of 128.546 µg/mL. For the other four plants, LC_50_ values ranged from lowest to highest as follows: 626.461, 1168.794, 1210.957 and 1368.340 µg/mL for *O. baccatus*, *E. arabicus*, *S. officinalis* and *P. crispa*, respectively.

Table 4 presents the LC_50_ values of the five tested extracts on the 2nd larval instar of *C. carnea*. The LC_50_ value of *P. penninervia* extract (232.095 µg/mL) was significantly lower than that of all other four plant species extracts according to relative median potency analyses (Table 5). For the other four plants, the LC_50_ values were 1137.564, 1299.649, 1448.547 and 1593.631 µg/mL for *P. crispa*, *O. baccatus*, *S. officinalis* and *E. arabicus*, respectively, and there was no significant difference among them according to relative median potency analyses. Meanwhile on aphid, the relative median potency analyses showed that all comparisons among five medicinal plants were significant except for *S. officinalis* versus *P. crispa* (RMP = 1.073, 95% CI: 0.834, 1.384) (Table 5). The comparison between the LC_50_ values of each plant extract for aphids and the green-lacewing larvae by relative median potency analyses indicated that *P. penninervia* (RMP = 0.516, 95% CI: 0.382, 0.665)*, O. baccatus* (RMP = 0.404, 95% CI: 0.287, 0.529) and *E. arabicus* (RMP = 0.692, 95% CI: 0.498, 0.924) were significantly more effective on *A. craccivora* than *C. carnea*. Meanwhile, the effects of *S. officinalis* and *P. crispa* were not significantly different to each other in LC_50_ values for both tested insects (RMP = 0.941, 95% CI: 0.736, 1.197 for *S. officinalis* and RMP = 0.692, 95% CI: 0.498, 0.924 for *P. crispa* (*A. craccivora* vs. *C. carnea*).

## 4. Discussion

The results indicate that even though the tested medicinal plant species belong to the same taxonomic family, their extracts contain different phenols and flavonoids and they have a different total content of these compounds. Similar results were reported for five endemic *Psiadia* species growing in Mauritius [26]. In another study, within three species of *Psiadia* genus, some components present in one were absent in the others [27]. The detection of different phytochemical constituents of the methanolic extract of *P. crispa* collected from Alkharj, Saudi Arabia indicated high amounts of phenols and flavonoids [28]. In Egypt, leaves of the same plant species (*Pulicaria undulata* (L.) C.A. Mey. sub sp. *undulata* = *P. crispa*) contained high levels of kaempferol, quercetin and caffeic acid [29] similar to the levels recorded in the present work. The flower of *Euryops pectinatus* was extracted with methanol and the total phenolic and flavonoid contents represented 49.41 and 23.37 µg/mg of the dried flower extract, respectively. The main flavonoids and phenolic acids detected were caffeic acid, quinic acid, protocatechuic acid, sinapic acid, chlorgenic acid, quercetin, kaempferol [30]. In our study, kaempferol and vanillic acid were found to be the major components in *E. arabicus* leaves but quercetin, caffeic acid and chlorgenic acid were not detected. Ethanol extraction of *O. baccatus* aerial parts from the Suez desert (Egypt) showed the presence of five quercetin and two kaempferol compounds with ethanol solvent [31] while in the present study, one compound from each of these was observed at an intermediate level.

In general, there are many factors that affect the total content and types of phenols and flavonoids extracted from plants, such as the extraction method, solvent type, plant origin, and the harvesting season. For example, leaf extracts of four species from the Asteraceae family, i.e., Psiadia arguta, Psiadia viscosa, Psiadia lithospermifolia and Distephanus populifolius with hexane, ethyl acetate, and methanol were studied for phenols and flavonoids contents. The content of phenolics varied from 24.05 to 231.6 mg gallic acid equivalent/g with the maximum level found in methanol extracts of P. arguta and D. populifolius whereas the highest flavonoids content was found in P. arguta methanolic extract with 65.7 mg quercetin/g [32]. Previous investigations of *E. arabicus* collected from the same region (Taif) and extracted with a mixture of CHCl3: MeOH (1:1, 4 L) using successive fractionation of the total extract on NP-Silica column, preparative thin layer chromatography (PTLC), followed by Sephadex LH-20, yielded only seven flavones [33], which were not detected in our study. The total flavonoid and phenolic content in aerial parts of *S. officinalis* collected from Eastern Serbia and Montenegro with chloroform, dichloromethane, ethanol and ethyl acetate extracts were significantly different and mostly depended on harvesting season and extraction solvent, although the plant origin had no effect on these compounds [34]. The butanol fraction of *S. officinalis* extract from Kalamazoo (USA) by ethanol solvent led to the isolation of 10 phenolic compounds including rosmarinic acid [35], which was detected in the same species in our study. Also, leaves of *S. officinalis* in New Zealand were extracted with 70% aq. acetone, then, the extract was fractionated on an HP20 column into water and methanol fractions, and led to the isolation of nine flavonoid and phenolic compounds including p-coumaric acid [36], which was detected in the same species in the present work. *Ochradenus arabicus* leaves and fruits from Oman were extracted with methanol followed by fractionation into ethyl acetate, chloroform, n-hexane, n-butanol and aqueous. The total flavonoid and phenolic contents were significantly higher in the sub-fraction of ethyl acetate [37]. Reports have stated that methanol as a solvent is more efficient in extracting flavonoids and phenols than other extraction solvents, i.e., distilled water, ethyl acetate, acetone, chloroform and hexane from the Datura metel plant leaves [38]. These compounds are more soluble in organic solvents like methanol and therefore, these solvents extract the largest amount of these compounds, which leads to greater insect mortality rates. The protectant and toxicity properties of botanical extracts may be due to the presence of secondary metabolites such as flavonoids, alkaloids, quinines and terpenoids. Over 9000 flavonoid compounds are known to exist in plants. They have a number of important functions in plants and can decrease digestibility, reduce their nutritive value, act as direct feeding deterrents or as toxins for insect pests [39]. Flavonoids are cytotoxic, interact with various enzymes through complexation and protect plants from insect pests by affecting their behavior, growth and development [40]. Aphid mortality may be due to contact toxicity or the initiation of some unknown physiological changes in the insect body [41].

In this study, the LC_50_ value of *P. penninervia* extract on aphids was the lowest among all plants (128.546 µg/mL), followed by *O. baccatus* (626.461 µg/mL) while *S. officinalis* and *P. crispa* had the highest LC_50_ values without any significant difference between them. Flavonoids inhibit the development of *A. craccivora* [42]. Although *A. craccivora* is one of nine aphid species associated with *S. officinalis* [43], the essential oil of *S. officinalis* at concentrations of 0.5, 0.3 and 0.1% caused mortality rates of 99.99, 96.65, 63.33% after 7 days on *A. craccivora* adults [44]. In the present study, the extract of *S. officinalis* had high LC_50_ value on *A. craccivora* compared to the other tested plant extracts, except for *P. crispa.* Water soluble compounds using ethanol extracts from 83 plant species from the Asteraceae family were highly variable in their toxicity against the larval mosquito, *Aedes fluviatilis* [45]. Also, the effect of three medicinal plant extracts belonging to Asteraceae family varied with the stage of insect, plant-derived material extract and exposure time while the methanol extracts had higher effects on larval growth inhibition, antifeedant properties and the mortality of *Tribolium confusum* [46].

Moreover, in this study, *P. penninervia* and *O. baccatus* extracts had the greatest amount of gallic acid compared to the other three plant extracts. The gallic acid extracted from the plant, *Terminalia nigrovenulosa* had significant nematicidal activity against *Meloidogyne incognita* in vitro [47]. Therefore, gallic acid in *P. penninervia* and *O. baccatus* extracts may be the cause of the high mortality for aphids.

Previous investigations have indicated that the extract of medicinal plant species has various effects on different insect species. For example, assessment of seven plant extracts for the toxic effects against four important pest insects from four different insect orders including the pea aphid, *Acyrthosiphon pisum* revealed that aphids were the most susceptible insect for the seven plant extracts tested with 100% mortality after 24 h. Moreover, although all the solvent fractions achieved high mortality in aphids, the butanol fraction was the most active against aphids. [21]. Meanwhile, the weakest insecticidal activity was shown by the fenugreek-based product [48]. However, the insecticidal activities of different six plant EOs against the cotton aphid, *Aphis gossypii* were strong but only two of them deserves attention due to the LD_50_ values were very close to the used standard Karate Zeon^®^ [49]. Also, insecticides based on different plant extracts showed not very good efficacy against the hop aphid, *Phorodon humuli* although the Pyrethrum insecticide, based on natural pyrethrins showed better action than azadirachtin based product NeemAzal T/S [50].

In this study, all five of the plant extracts were more toxic against the aphid, *A. craccivora,* than *C. carnea* lacewing, but only three species produced significant differences. Also, the LC_50_ value of *P. penninervia* extract on the 2nd larval instar of *C. carnea* (232.095 µg/mL) was significantly lower than those of all other four plant species extracts, meanwhile the other four plants did not show significant difference among them according to relative median potency analyses. On the other hand, the most significant aphid mortalities were observed (lower LC_50_ values) for *O. baccatus*, *E. arabicus* and *P. penninervia* extracts. Moreover, extracts from these three plants were significantly more effective on *A. craccivora* with less impact on the predator *C. carnea*, causing significantly less mortality (RMP = 0.404, *A. craccivora* vs. *C. carnea*) for *O. baccatus* compared to *E. arabicus* and *P. penninervia* extracts. The plant extracts from the Asteraceae family were nontoxic to *C. carnea* and *Coccinella undecimpunctata* when they are used to control the cabbage aphid, *Brevicoryne brassicae* [51]. Extracts of nine Ghanaian plants controlled the cabbage aphid, *Brevicoryne brassicae* as effectively as the synthetic insecticide, emamectin benzoate but were significantly less harmful to ladybirds [52]. In general, the plant-based products demonstrate effective pesticide activity but also pose threats to beneficials and other non-target insects such as natural enemies and pollinators [18,53,54,55,56]. According to our findings, *O. baccatus* extract had a high impact on aphids and was safer for the predator. This suggests that *O. baccatus* extract could be used for aphid control in combination with *C. carnea*. Therefore, the integration of some plant extracts with *C. carnea* in IPM strategies could be efficient. For example, the efficacy of six plant extracts belonging to three families and including *Pulicaria incisa* against *A. fabae* and the 2nd larval instar of two predators, *C. carnea* and *C. undecimpunctata*, indicated that all the mortality rates for aphids were significantly different than the control. However, for the two tested predators, there were insignificant differences between the tested extracts and the control. Moreover, in the field, all the tested plant extracts were shown to be efficient in controlling the aphid without harmful effects on the predators [57]. Therefore, experiments in the field should be conducted for *O. baccatus* extract to estimate its effect on aphids and associated predators.

## 5. Conclusions

*O. baccatus* extract phytochemicals may provide a new set of treatments for further development as biorational-based insecticides against plant-feeding hemipterans, like aphids. Other investigations could be carried out to further examine the effects of these plant extracts, especially *O. baccatus* extract, on other instars or stages of *C. carnea*, other beneficial insects and other piercing sucking insects in order to estimate the effects on these insect pests and their predators.

## Figures and Tables

**Table 1:** The five medicinal plants used in the present study.

No.	Scientific Name	Common Name	Family Name
1	*Psiadia penninervia*	Lakuna, Pisidic tribes	Asteraceae (Compositae)
2	*Salvia officinalis*	Garden sage, Common sage, or Culinary sage	Lamiaceae
3	*Ochradenus baccatus*	Taily weed	Resedaceae
4	*Pulicaria crispa*	Dhola lizru	Asteraceae (Compositae)
5	*Euryops arabicus*	Djibouti	Asteraceae (Compositae)

**Table 2:** Components of phenols and flavonoids in the five medicinal plant extracts (mg/kg).

Compounds	*Psiadia penninervia*	*Salvia officinalis*	*Ochradenus baccatus*	*Pulicaria crispa*	*Euryops arabicus*
Gallic acid	3.14981	1.95416	6.73492	3.71654	ND
Catechol	18.58176	37.61870	5.87161	3.35947	56.47470
p-Hydroxy benzoic acid	23.41773	ND	23.37553	7.97348	39.50618
Catchin	2.86307	4.91904	1.48770	2.84388	2.14091
Chlorgenic acid	4.53216	7.56331	ND	10.66796	ND
Vanillic acid	408.63805	4.82321	91.62633	337.59454	653.50251
Caffeic acid	ND	3.72419	ND	6.63623	ND
Syringic acid	2.83148	8.65167	1.69567	11.54246	21.80036
p-Coumaric acid	11.65656	1.13434	1.28208	36.73665	37.64450
Benzoic acid	104.93644	ND	41.55768	155.18990	118.06012
Ferulic acid	11.75697	174.80431	1.65414	17.19678	9.99849
Rutin	102.00465	68.54289	60.39530	96.12466	25.04535
Ellagic	11.97470	227.49873	10.27533	168.42101	1.80337
o-Coumaric acid	9.54869	5.13542	10.34360	7.09154	12.84780
Resvertol	108.51695	508.72487	388.57594	349.52147	266.34312
Cinnamic acid	ND	15.46662	6.50717	55.39434	7.19120
Quercetin	28.57828	26.80540	18.90163	82.79101	ND
Rosmarinic acid	19.25397	9.83388	35.70374	ND	294.95133
Neringein	ND	11.29797	ND	148.58088	ND
Myricetin	ND	10.58877	11.00759	72.89414	ND
Kaempferol	ND	4.26682	11.03976	102.88033	478.60399
Total	869.40644	1125.56781	721.36812	1667.83976	2025.91391

ND: Not detected.

**Table 3:** Values of LC_50_ (µg/mL) for extracts from five medicinal plant species against adults of *Aphis craccivora*.

Plant Extract	LC_50_ (CI limits)	Intercept ± SE	Slope ± SE	ꭓ^2^
*Psiadia penninervia*	128.546 (97.120–156.662)	−4532 ± 0.638	2.149 ± 0.264	0.374
*Salvia officinalis*	1210.957 (982.616–1668.270)	−7.628 ± 0.937	2.474 ± 0.337	1.108
*Ochradenus baccatus*	626.461 (542.038–745.145)	−6.587 ± 0.646	2.35 ± 0.242	0.465
*Pulicaria crispa*	1368.340 (1043.539–2127.691)	−6.179 ± 0.287	1.970 ± 0.785	4.482
*Euryops arabicus*	1168.794 (893.076–1785.935)	−5.232 ± 0.648	1.706 ± 0.242	0.162

CI: Confidence Interval limits.

**Table 4:** Values of LC_50_ (µg/mL) for extracts from five medicinal plant species against 2nd larval instar of *Chrysoperla carnea.*

Plant Extract	LC_50_ (CI limits)	Intercept ± SE	Slope ± SE	ꭓ^2^
*Psiadia penninervia*	232.095 (190.242–274.221)	−4.452 ± 0.542	1.882 ± 0.215	0.822
*Salvia officinalis*	1448.547 (1131.509–1714.452)	−6.788 ± 0.873	2.148 ± 0.316	6.405
*Ochradenus baccatus*	1299.649 (1073.062–1789.167)	−9.761 ± 1.404	3.135 ± 0.492	3.130
*Pulicaria crispa*	1137.564 (979.468–1432.116)	−11.026 ± 1.516	3.608 ± 0.529	1.789
*Euryops arabicus*	1593.631 (1154.401–2791.251)	−5.770 ± 0.774	1.802 ± 0.284	4.789

CI: Confidence Interval limits

**Table 5:** Relative susceptibilities of 2nd larval instar of *Chrysoperla carnea* and adults of *Aphis craccivora* to methanol extracts from five medicinal plants species.

Plant Extract	*Psiadia penninervia*	*Salvia officinalis*	*Ochradenus baccatus*	*Pulicaria crispa*	*Euryops arabicus*
*Psiadia penninervia*		**5.793**	**7.070**	**6.518**	**5.345**
*Salvia officinalis*	**0.092**		1.220	1.125	0.923
*Ochradenus baccatus*	**0.195**	**2.112**		0.922	0.756
*Pulicaria crispa*	**0.099**	1.073	**0.508**		0.820
*Euryops arabicus*	**0.129**	**1.402**	**0.664**	**1.307**	

Relative median potency analyses (RMP) values of the comparisons: *A. craccivora* (Lower left of table), *C. carnea* (Upper right of table). Each value indicates the comparison of plant in the column versus the plant in the row. Values < 1 indicate more susceptibility; Values > 1 indicate less susceptibility. Bold values indicate significant values (95% CI ≠1).
